# Anemia management in non-menopausal women in a primary care setting: a prospective evaluation of clinical practice

**DOI:** 10.1186/s12875-020-1086-5

**Published:** 2020-01-21

**Authors:** Sabine Bayen, Charline Le Grand, Marc Bayen, Florence Richard, Nassir Messaadi

**Affiliations:** 1 Department of General Practice, University of Lille (F), Pôle formation Faculté de médecine Henri Warembourg, 59045 Lille CEDEX 1, France; 2 Department of Medical Pharmacology & Neurology, INSERM UMRS 1171, University of Lille (F), Lille, France; 3 Department of Epidemiology and Public Health, INSERM UMRS 1167, University of Lille (F), Lille, France; 4 SCALab CNRS UMR 9193, University of Lille, Lille, France

**Keywords:** Primary care, Evaluation of clinical practice, Anemia management, Non-menopausal, Non-pregnant, Women, Iron deficiency

## Abstract

**Background:**

The study aimed to analyze anemia management in non-pregnant, and non-menopausal women aged from 18 to 50 years old, in a French primary care setting.

**Methods:**

An observational descriptive prospective study was conducted between November 2018 and February 2019. Inclusion criteria were as followed: anemia diagnosed in women aged from 18 to 50, not pregnant and not menopausal. Quantitative and qualitative data were anonymized and collected through an electronic survey. Investigating general practitioners completed the questionnaire for each newly diagnosed woman. Mean values and medians were calculated for the quantitative data. Answers to the open questions were encoded manually and proportions of the different modalities have been calculated.

**Results:**

Altogether, 43 women with anemia were ascertained. Moderate microcytic anemia, due to an iron deficiency in a context of menorrhagia, was the most observed anemia profile. The mean value of hemoglobin was 10.5 ± 1 g/dl. Among these women: 32 (74%) presented an iron deficiency, 17 (53%) had inappropriate intakes, and 9 (28%) reported menorrhagia. For 17 (40%) women, unnecessary or inappropriate exams were prescribed. The investigations did not allow to establish a differential diagnosis for 12 women (28%). Even for similar clinical situations, anemia management was variable. Among the women who presented iron deficiency, 15 (47%) were informed about an iron-rich diet and received a daily iron supplementation of ferrous sulfate between 80 mg and 160 mg.

**Conclusions:**

Our study highlights that, in the absence of specific national guidelines for anemia management in non-pregnant, non-menopausal women in primary care settings, French GPs undergo various clinical management strategies leading to a heterogeneous, sometimes inappropriate follow-up.

Women with iron deficiency were prescribed higher daily iron supplementation than recommended, according to new evidence, suggesting a maximal daily dose of 50 mg of elementary iron in a context of Hepcidin up-regulation in the case of an iron overload.

Additional longitudinal studies with a bigger sample size and randomized controlled trials are needed to confirm our results and to elaborate national guidelines.

## Background

The World Health Organization (WHO) defines anemia in women as a hemoglobin concentration below to 12 g/dl [1]. In 2016, the WHO estimated the prevalence of anemia in women of childbearing age at 30.2% worldwide, at 20.2% in Europe, and at 18.1% in France [1]. The WHO aims to decrease the rate of anemia in this population by 50% by 2025 [2]. Fifty per cent of anemia cases are due to iron deficiency [3] which is the most frequent nutritional deficiency and concerns a third of the population worldwide [4]. In France, the National Nutrition and Health Survey (ENNS) 2006–2007 revealed that 8.7% of women present an iron deficiency (ID). Women at the age between 18 and 29 years old, are the most concerned subpopulation: 17.2% of this subpopulation of women present deficiencies [5].

Anemia is considered as an independent risk factor of morbid mortality regardless of age or gender [6]. Its consequences on health are extent and potentially serious: anemic syndrome, decrease of quality of life [7, 8], decrease of physical [9–11], and mental capacities [12–14], psychiatric disorders [15–17], vulnerability to infections [18, 19], and dander disorders [20].

The majority of anemia cases are diagnosed and managed in primary care [21]. Despite this fact, studies about anemia management in primary care are still underrepresented. Nowadays, French General Practitioners (GPs) have few guidance notes for daily clinical anemia management in non-menopausal women. But no national guidelines exist for anemia management in non-pregnant, non -menopausal women who are followed-up in a French primary care setting. The main goal of our study was to describe how GPs manage anemia in that population. Moreover, we looked for possible similar practices among GPs and described the etiologic profiles of anemia observed in our sample.

## Methods

We conducted an exploratory study in a primary care setting to analyze GP’s anemia management in non-menopausal women between 18 and 50 years old, non-pregnant, from diagnosis to treatment in the case of iron deficiency anemia (IDA). The study was observational, descriptive, prospective, and conducted between November 12th, 2018 and February 10th, 2019.

The inclusion criteria for the investigating GPs were as followed: Being a GP surrogate, or an installed GP, working in the following French regions: *Hauts-de-France* and *Pays de la Loire*. GPs were randomly selected from the telephone directory in each region in order to include a number between 30 and 40 investigators. The GPs were then individually informed about the study protocol, first by e-mail and followed by a phone call if necessary, some of them were met physically. Their socio-demographic characteristics were assessed: age, gender, region, and terms of exercise, such as urban or rural area, and working as a GP or a surrogate.

The data were collected and anonymized through an auto-administered questionnaire created by the Lime Survey® software. Each investigating GP completed a questionnaire for each new included woman presenting anemia. The GPs had access to their questionnaire via a code to view complementary exam results, and treatments related to the anemia management. Each GP had the possibility to complete 5 patient files. Before the study started, the questionnaire was tested by 6 GPs. The questionnaire included 5 questions: “initial biological data”, “initial demographical and clinical data”, “terms of prescription of biological exams”, “your decision and action at the moment of results’ reception “, and “in case of complementary exams, or specialized expert opinion”. Inclusion criteria for patients were as followed: age between 18 and 50 years old, non-menopausal, newly diagnosed anemia (according to the WHO criteria). Pregnancy was a non-inclusion criterion. To encourage the GPs’ participation in the study, they were called by phone every 3 weeks. The questionnaire data were collected and transcribed in an Excel sheet. Quantitative data were calculated as means and averages. Qualitative data, (free answers to the open questions) were encoded by a global content analysis. New categories were created manually during the data collection along with the needs, according to the answers which were progressively returned by the GPs. We calculated the percentage proportion of the present or absent different terms using the *EPI info* software. We used non parametrical tests in the context of a small sample: * Kruskall-Walis test, and* fixed IC at *95%* (*p* < 0.05).

## Results

### The process of data collection

(cf. Figure 1).

**Fig. 1:**
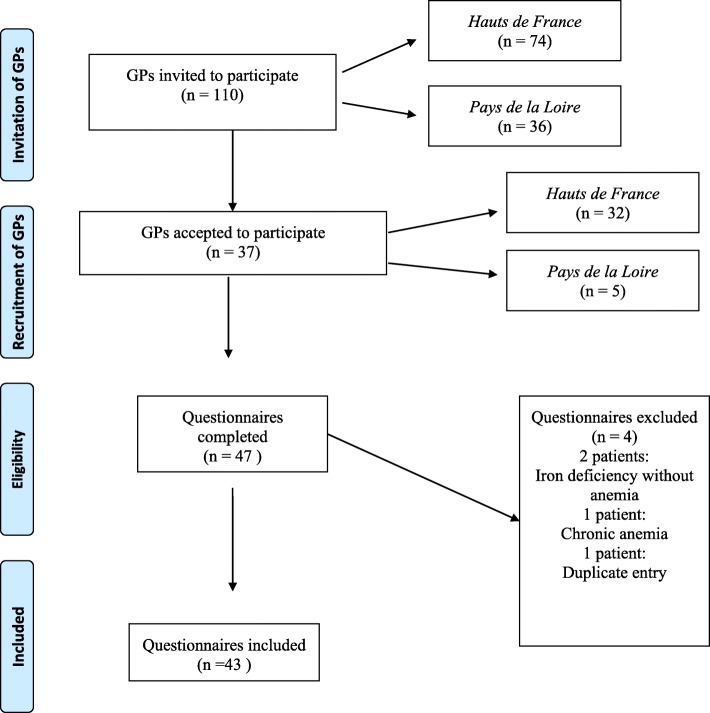
Flow-Chart of data collection (page 6, line 116)

### The GPs’ profiles

The average age of the GPs was 39 years old, and 19 (50%) were women. Thirty GPs (81%) worked in an urban area, and 17 (46%) had their own office. The women’s blood test prescriber was for 35 their GP (82%) or the GP surrogate, and for 7 (16%) another substitute. One woman declared her gynecologist as her prescribing physician. Initial symptoms leading to the blood test prescription were asthenia 72% (*n* = 31), pallor 19% (*n* = 8), menstruation disorders 12% (*n* = 5), dander disorders (alopecia or brittle nails) 9% (*n* = 4), and anxious-depressive syndromes 5% (*n* = 2). Anemia was diagnosed during a routine check-up, a pre-therapeutic evaluation, or follow-up for 12% (*n* = 5) of the women.

### The patients’ profiles

Fourteen per cent (*n* = 6) of the patients were between 18 and 25 years old, 35% (*n* = 15) between 26 and 33 years, 25.5% (*n* = 11) between 34 and 41 years old, and 25.5% (*N* = 11) between 42 and 50 years old. Thirty-three per cent (*n* = 13) had no medical history. Twenty-three per cent (*N* = 10) had prior cardiovascular history, 14% (*n* = 6) had psychiatric disorders, and 12% (n = 5) had a prior gynecological history. Thirty-seven per cent (*n* = 16) took no medication. Twenty-one percent (*n* = 9) were taking a birth control pill (40% (*n* = 6) of the 26–33 years old women).

The average hemoglobin blood level was 10.5 g/dl (± 1), mean: 10.6 g/dl (10; 11). The average mean corpuscular volume (MCV) was 81μm^3^ (± 8.6). Anemia was estimated mild in 40% (*n* = 17) of the cases, moderate in 58% (*n* = 25) of the cases, and severe in 2% (*n* = 1) of the cases. We did not observe any significant differences neither regarding the hemoglobin level nor the severity of anemia according to the different age groups (*p* = 0.41, and *p* = 0.78).

### Anemia profiles

**Fig. 2:**
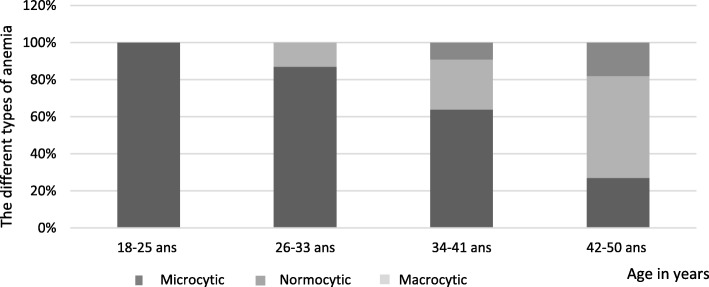
The different types of anemia (microcytic/normocytic/macrocytic) (page 7, line 144)

Sixty-seven per cent (*n* = 29) of the cases of anemia were microcytic, 26% (*n* = 11) normocytic, and 7% (*n* = 3) macrocytic. There were no significant differences between the MCV according to the different age groups (*p* = 0.07). However, the distribution of the three forms of anemia: (microcytic, normocytic and macrocytic) varied in a significant way according to the different age groups (*p* = 0.003) (cf. Figure 2).

### The organization of the patients’ follow-up

Ninety-three per cent (*n* = 40) of the patients came for a follow-up appointment, one patient had a phone call, and two patients did not come to their appointment. When the patients were not available for a consultation when the GP received the blood test results, the GP did not call the patient to inform them immediately in 44% (*n* = 11) of the cases. The reasons were: to encourage the patient’s autonomy in 36% (*n* = 4) of the cases, and the absence of urgent nature of the result in 90% (*n* = 10) of the cases. The patients were contacted in 56% (*n* = 14) of the cases: in order to inform, and reassure them in 64% (*n* = 9) of the cases, to evaluate their clinical tolerance in 21% (*n* = 3) of the cases, to prescribe complementary exams in 28% (*n* = 4) of the cases, or to inform the patient that a treatment was necessary in 43% (*n* = 6) of the cases.

After receiving the blood test results, a clinical exam was performed in 74% (*n* = 32) of the cases: 84% (*n* = 27) of these clinical exams were a quantification of menstruation, 31% (*n* = 10) were a gynecological exam, and 81% (*n* = 26) were an evaluation of the cardiological tolerance and/or an exam of the digestive system. The GPs initially based their diagnosis on other elements of the complete blood count (CBC) in 49% (*n* = 21) of the cases and identified an eventual regenerative character of the present anemia in 30% (*n* = 13) of the cases.

### Additional exams

An additional biological exam was prescribed in 49% (*n* = 21) of the cases (cf. Table 1). For 40 % (*n* = 17) of the patients, the prescribed exams were not indicated. The vitamin B12 and B9 blood levels were prescribed respectively in 35% (*n* = 15), and 37% (*n* = 16) of the cases. These dosages were not justified in 67% (*n* = 10), and 69% (*n* = 11) of the cases. In fact, B12 and B9 blood levels were physiological standard in 100% of the cases, or their dosages were not recommended. In case of justified prescriptions, their serum levels were low for B12 in 20% (*n* = 1) of the cases and for B9 in 40% (*n* = 2) of the cases. The serum level of Thyroid Stimulating Hormone (TSH) was prescribed in association to the anemia blood test in 17% (*n* = 7) of the cases, which was never recommended. Finally, the TSH was physiological standard in 100% of the cases. (Table 1).

**Table 1:** Prescription of additional exams according to the type of anemia (page 8, line 171)

Type of anemia	Ferritinemia	TSH	B9	B12	Creatinine
Microcytic	73% (*n* = 11)	40% (*n* = 6)	87% (*n* = 13)	80% (*n* = 12)	13% (*n* = 2)
Normocytic	25% (*n* = 1)	25% (*n* = 1)	25% (*n* = 1)	25% (*n* = 1)	0%
Macrocytic	100% (*n* = 2)	0%	100% (*n* = 2)	100% (*n* = 2)	50% (*n* = 1)
All types	67% (*n* = 14)	33% (*n* = 7)	76% (*n* = 16)	71% (*n* = 15)	14% (*n* = 3)

An expert opinion was requested in 37% (*n* = 16) of the cases. The gastroenterologists and gynecologists were solicited in 44% (*n* = 7) for an expert opinion each, and a hematologist, and a digestive surgeon were solicited, once each. All requested expert opinions were motivated by an organ specific symptomatology and were completely justified.

### Etiologies of anemia

The etiologies of anemia were: iron deficiency 77% (*n* = 33), folate deficiency 5% (*n* = 2), vitamin B12 5% (*n* = 2), dysthydroidism 2% (*n* = 1), cirrhosis 2% (*n* = 1), heterocygote β-thalassemia 2% (*n* = 1), hemolytic anemia *Minkowski Chauffard* 2% (*n* = 1). The frequency of etiologies according to profiles of anemia are summarized in Table 2.

**Table 2:** MCP and severity of anemia according to profiles of anemia (page 8, line 180)

Etiology	Profile of anemia according to MCV			Profile of anemia cases and severity			All anemia
	Microcytic	Normocytic	Macrocytic	Mild	Moderate	Severe	
Iron Deficiency	72% (*n* = 24)	25% (*n* = 8)	3% (*n* = 1)	41% (*n* = 13)	56% (*n* = 18)	3% (*n* = 1)	74% (*n* = 32)
Dysthyroidie	0%	0%	100% (*n* = 1)	0%	100% (*n* = 1)	0%	2% (*n* = 1)
Cirrhosis	0%	0%	100% (*n* = 1)	0%	100% (*n* = 1)	0%	2% (*n* = 1)
Kidney failure	0%	0%	100% (*n* = 1)	0%	100% (*n* = 1)	0%	2% (*n* = 1)
Vitamin B12 Deficiency	0%	100% (*n* = 2)	0%	50% (*n* = 1)	0%	50% (*n* = 1)	5% (*n* = 2)
Vitamin B9 Deficiency	0%	0%	100% (*n* = 2)	0%	100% (*n* = 1)	50% (*n* = 1)	5% (*n* = 2)
Heterocygous Β-thalassemia	100% (*n* = 1)	0%	0%	0%	100% (*n* = 1)	0%	2% (*n* = 1)

Among the IDA: 72% (*n* = 23) were microcytic, 25% (*n* = 8) normocytic, and 3% macrocytic (*n* = 1). Among the 9 cases of non-microcytic anemia: 2 of them were associated to a vitamin B12 deficiency, 1 was associated to kidney failure and vitamin B9 deficiency, and in 6 other cases the biological explorations did not allow the physician to exclude an intrinsic origin. The biological explorations were insufficient to judge if a single etiology was responsible for anemia in 28% (*n* = 12) of the cases. Ferritin was dosed in 84% (*n* = 36) of the cases, and that even before the diagnosis of anemia in 51% (*n* = 22) of the cases. The average Ferritin level in the blood was 9.5 μg/l (± 9.4), and the mean was 10.6 (5; 10). Seventy-four per cent of the patients presented an ID. Regarding the serum iron reserve: 2.3% were low, 7% were normal, and 16% among them had no Ferritin dosage. Five IDA were diagnosed in absence of a Ferritin dosage, and 4 diagnosis were based on a low iron blood level. One anemia was diagnosed despite of a normal Ferritin blood level. In these cases, the diagnosis of ID was not retained.

The observed serum iron reserves according to severity of anemia and age groups are summarized in Table 3.

**Table 3:** Serum iron reserves according to severity of anemia and age groups (page 9, line 194)

Serum iron reserve		Iron deficiency	Low reserve	Normal reserve	Ferritin not dosed
Type of anemia according to MCV	Microcytic	79% (*n* = 23)	0%	3% (*n* = 1)	17% (*n* = 5)
	Normocytic	72% (*n* = 8)	9% (*n* = 1)	9% (*n* = 1)	9% (*n* = 1)
	Macrocytic	33% (*n* = 1)	0%	33% (*n* = 1)	33% (*n* = 1)
Type of anemia according to severity	Mild	76% (*n* = 13)	0%	6% (*n* = 1)	18% (*n* = 3)
	Moderate	72% (*n* = 18)	4% (*n* = 1)	12% (*n* = 2)	16% (*n* = 4)
	Severe	100% (*n* = 1)	0%	0%	0%
Age groups	18–25 years	83% (*n* = 5)	0%	0%	17% (*n* = 1)
	26–33 years	87% (*n* = 13)	0%	0%	13% (*n* = 2)
	34–41 years	63% (*n* = 7)	0% (*n* = 1)	9% (*n* = 1)	27% (*n* = 3)
	42–50 years	63% (*n* = 7)	9% (*n* = 1)	18% (*n* = 2)	9% (*n* = 1)

The biological etiological assessment was complete in 59% (*n* = 19) of the cases of iron deficiency. Seven gastric fibroscopies (GF) were performed, and 86% (*n* = 6) justified: 83% (*n* = 5) because of digestive symptoms or digestive medical histories, and 17% (*n* = 1) by the severity of anemia. Eight GF were indicated (7 because of anemia < 10 g/dl, and 1 because of digestive symptoms), but not realized. Two indicated coloscopies were realized, for which the results were normal. Five, pelvic ultrasounds, were performed and justified by present menstruation disorders. Four indicated pelvic ultrasounds were not performed.

### Etiologies of iron deficiency

Iron deficiency was associated to bleeding in 26% (*n* = 11) of the cases: menorrhagia in 82% (*n* = 9) of the cases, (3 cases of fibroma, 1 functional bleeding, 1 endometriosis, and 4 unexplored cases of menorrhagia), digestive bleeding in 18% (*n* = 2) of the cases: (1 esophagitis and 1 benign gastric ulcer due to Helicobacter pylori). Two cases of ID were associated to malabsorption (1 due to a by-pass and 1 due to an already diagnosed celiac disease). Coeliac disease was never investigated in this primary care setting, except once after a gastroenterological expert opinion, but coeliac disease was not diagnosed.

### Iron deficiency management

After evaluating the patient’s nutritional status, GPs noticed a low iron intake in 53% (*n* = 17) of the cases of ID, and especially for 2 women who followed a restrictive diet. Eighty-eight percent of the patients (*n* = 38) took an iron supplementation. When the first blood test results were received, 70% (*n* = 30) of the patients were prescribed an iron medical supplementation. The supplementation was not indicated in 33% (*n* = 10) of the cases because ID was not proven. After the reception of additional exams, eight new patients were supplemented, which was justified because the ID was confirmed. The ferrous sulfate was the most prescribed molecule and concerned 89% of the patients (*n* = 34).

The daily dose of iron was < 80 mg/d in 26% (*n* = 10) of the cases, between 80 and 160 mg in 42% (*n* = 16) of the cases, > 160 mg/d in 18% (*n* = 7) of the cases, and the dose was not specified in 13% (*n* = 5) of the cases. An associated supplementation was prescribed in 16% of the cases of ID. Vitamin C was associated in 11% (*n* = 4) and vitamin B9 was in 5% (*n* = 4) of the cases. The initial treatment duration was 3 months in 71% (*N* = 27) of the cases, 1 month in 11% (*n* = 4) of the cases, 6 months for a single patient. Forty-one per cent (*n* = 15) of the patients received information by their GP. An iron-rich diet was advised in 93% (*n* = 14) and explanations about inhibitors of iron intake, such as tea, were given in 20% (*n* = 3) of the cases.

## Discussion

In France, few official guidance notes exist for ID and IDA management. These guidelines don’t target anemia management for non-pregnant, non-menopausal women in primary care settings.

The high authority of health (HAS) published a report in 2011 to remind healthcare professionals of the rational choice of exploring of patient’s biological iron status [22]. In 2008, prescriptions of serum iron level in blood tests (isolated or in association with other dosages) represented 39% of biological exams aiming to explore an iron status. In our sample, this dosage was performed in 19% of the cases. However, GPs were younger, and the proportion of women was higher than in current French GP populations [23]. It would be interesting to conduct a sample-based study of clinical practice. In contrast, the ID management has led to heterogeneous prescriptions, especially regarding supplementation posologies. The daily recommended dose of iron for adults varies between 60 and 200 mg of elementary iron [24–26]. Nevertheless, some studies suggest that a daily dose of iron superior to 60 mg, decreases the absorption the following day as a consequence of the increase of Hepcidin. Indeed, this peptide plays a major role in the regulation (diminishing) of intestinal iron intake [27]. In case of ID, Hepcidin output is decreased to increase the blood iron level. At the opposite, an iron overload increases the output of Hepcidin. Therefore a daily administration of 15 mg and 50 mg of elementary iron proved to be as effective as one dose of 150 mg for elderly patients presenting ID [28, 29]. In our study, only one iron supplementation prescription was inferior to 60 mg. That is not surprising, considering that the recent findings were not included in the few existing official guidelines.

Regarding anemia management, our participating GPs were self-directed. The etiological assessment was correctly conducted and succeeded to a correct etiological diagnosis in 72% of the cases. When expert opinions were solicited, they were justified and led to changings in clinical management. At the opposite, some prescribed complementary exams did not seem appropriate. Moreover, the prescription of useless exams for frequent conditions such as anemia present a significant additional cost. Nowadays, the supervision of public costs related to health systems is increasing. A Spanish study estimates the annual extra cost due to inappropriate complementary exams during anemia clinical management in a primary care setting at 6 million euros [30]. The absence of recommendations in primary care to manage anemia may explain the exhaustive inappropriate prescriptions. Most of the anemia are diagnosed and managed in a primary care setting. Another Spanish observational transversal study [21] in a primary care setting revealed that over 1 year 152 new cases of anemia were diagnosed, of which 73% during primary care consultations. It is crucial to know the characteristics of anemia which are prevalent in primary care to guide their diagnosis and select relevant complementary exams. But nowadays, few studies were conducted in a primary care setting. Current literature consists of retrospective studies conducted in a hospital setting [31, 32]. These results are not applicable to a primary care setting. To promote research in primary care, real life data must be generated in a primary care setting. In industrialized countries, despite the abundant supply of food, many people develop ID due to a lack of alimentary intake. Iron cannot be synthesized by the organism, and its persistent quantity is extremely unstable. The daily loss of iron is due to: epithelium desquamation, bile loss, sweating, or bleeding [33]. For women, an additional iron loss is due to menstrual bleeding. Therefore, women are exposed to a higher risk of iron deficiencies than men. An American study of 2009 has evaluated the daily iron excretion: 1.18 mg for men, and 1.58 mg for women during menstruation [34]. Compensating these losses by an appropriate diet is essential. In France, the average dietary intake of iron is inferior to the current nutritional recommendations for non-menopausal women [35, 36]. Addressing iron rich diet consumption during an appointment with our patients may improve primary prevention, decrease recurrences, and improve ID treatment. Various dietary interventions are already available to treat ID and IDA [37]. The combination of these measures seem to improve the iron status [38–41]. New technologies, for instance the HemoScreen™ instrument, may favor and facilitate anemia management in a primary care setting because it allows a quick and correct evaluation of the red blood cell parameters [42].

Strengths: Our study is original because the design was prospective, and GPs participated in their real-life primary care setting which provided insight into their authentic clinical practice.

Limits: To encourage the investigating GPs to participate in that study, during which their clinical practice was evaluated, an anonymous online questionnaire was used to include the women. Therefore, we can’t deduce the number of enrolled women by each GP. Few statistical tests were conducted because of the small sample size due to the prospective recruitment and the small number of returned completed questionnaires. The obtained results, thanks to the statistical tests used, are acceptable only for our sample.

## Conclusion

Moderate microcytic anemia, due to an iron deficiency in a context of menorrhagia, was the most observed anemia profile. Less than a half of the women were informed about iron-rich-diet and received a daily iron supplementation of ferrous sulfate between 80 mg and 160 mg which is not recommended according to new evidence, suggesting a maximal daily dose of 50 mg of elementary iron in a context of Hepcidin up-regulation in the case of an iron overload.

Our study highlights that, in the absence of precise national guidelines for anemia management in non-pregnant, non-menopausal women in primary care settings, French GPs undergo various clinical management strategies leading to a heterogeneous, sometimes inappropriate follow-up.

Additional longitudinal studies with a bigger sample size and randomized controlled trials are needed to confirm our results and to elaborate national guidelines.

## Data Availability

The data sets used and/or analyzed during the current study are available from the corresponding author on request.

## Abbreviations

CBC: Complete blood Count
CI: Confidence interval
GP: General Practitioner
HCT: Hematocrit
HGB: Hemoglobin
ID: Iron Deficiency
IDA: Iron Deficiency Anemia
MCH: Mean cell hemoglobin
MCHC: Mean cell hemoglobin concentration
MCV: Mean corpuscular volume
POCT: Point of care testing
RBC: Red blood cells
WBC: White blood cells
WHO: World Health Organization
